# Whether age of menarche is influenced by body mass index and lipoproteins profile? a retrospective study

**Published:** 2012-07

**Authors:** Maryam Farahmand, Fahimeh Ramezani Tehrani, Fereidoun Azizi

**Affiliations:** 1 *Reproductive Endocrinology Research Center, Research Institute for Endocrine Sciences, Shahid Beheshti University of Medical Sciences, Tehran, Iran.*; 2 *Department of Obstetrics and Gynecology, Reproductive Endocrinology Research Center, Research Institute for Endocrine Sciences, Shahid Beheshti University of Medical Sciences, Tehran, Iran.*; 3 *Department of Endocrinology, Endocrine Research Center, Research Institute for Endocrine Sciences, Shahid Beheshti University of Medical Sciences, Tehran, Iran.*

**Keywords:** *Age of menarche*, *Body Mass Index*, *Serum lipid profile*

## Abstract

**Background:** Menarche, a milestone in the reproductive life span of a woman, is influenced by several genetics and environmental factors. There is no consensus regarding the impact of body mass index (BMI) and lipid profiles on the age of menarche, as the results of various studies demonstrate.

**Objective: **To investigate the correlation between age of menarche and BMI/lipoprotein profile in a community sample of Iranian girls.

**Materials and Methods:** In the study, 370 girls, aged 10-16 years, who began their menarche within six months prior to the study, were recruited from the Tehran Lipid and Glucose Study (TLGS) population. Information was documented regarding their body composition, including height, weight, BMI, waist and hip circumference were collected and their lipid profiles were assessed after a 12-hour fast.

**Results:** In this study, the mean±SD of age of menarche and BMI were 12.6±1.1 years and 21.7±3.9 kg/m^2^, respectively. There were statistically significant relationships between age of menarche and height, BMI, waist circumference, and the maternal educational level. The relationship between age of menarche and the weight and lipid profiles of subjects was not statistically significant.

**Conclusion:** Age at menarche is not influenced by lipid profiles but it is influenced by BMI.

## Introduction

Puberty marks an especially important stage of life with significant physical, mental and emotional changes. The onset of puberty reflects numerous health aspects of a population including the timing of sexual maturation, growth and nutritional status and environmental conditions, in well-nourished, healthy girls and menarche is largely dependent on genetics, as confirmed by studies on the menarcheal age of twins, sisters and mother-and-daughter pairs (1-9). 

The impacts of anthropometric parameters, central obesity indexes and body fat have been investigated by several studies but have yielded different results (9-11). In a study by Garcia et al, there was no correlation between age at menarche and BMI (10), while other studies report contradictory findings i.e. the age at menarche among girls with higher BMI was less than those girls with lower BMI (12-16). Various endocrine factors affect the rate of puberty and the gathering of body fat (17, 18). 

There are limited studies in literature on the correlation between lipid profiles and age at menarche. We aimed to assess the correlation between age at menarche and anthropometric parameters and lipid profiles among a community based sample of girls whose menarcheal onset had occurred during the six months prior to the study. 

## Materials and methods

Our cases were recruited from the Lipid and Glucose study (TLGS) cohort, an ongoing prospective study, initiated in 1998, with the aim of determining the prevalence of non-communicable disease risk factors. The TLGS includes 15,005 people, ≥3 years selected from a geographically defined population using multistage cluster sampling. In this study the subjects were selected from the TLGS second phase between 2002-2005 years. Inclusion criteria were: 1) Age range 10-16 years. 2) Beginning of menarche within the six months prior to the study. And those with any history of chronic disorders (such as: diabetic mellitus, chronic heart disease) were excluded. Demographic and lifestyle variables were collected through face-to-face interviews by trained interviewers. 

The follow-up included a general physical examination, height and weight measurements, their blood samples were taken after 12-hours over night fasting and lipid profile analyses were done at the TLGS research laboratory on the day of blood collection. The onset of menarche was asked and the date of the first cycle was recorded. The mother’s educational level was categorized to less than high school diploma, high school diploma and more than high school diploma. Weight was measured to the nearest 0.1 kg on a calibrated beam scale. Height and waist circumference (WC) were measured to the nearest 0.5 cm with a measuring tape, WC was measured midway between the lower rib margin and the iliac-crest at the end of a gentle expiration. Body mass index was calculated as weight in kilograms divided by the height in meters squared (kg/m^2^) (20).

BMI was categorized, according to WHO guidelines, into four groups. Those with BMI <15^th^ percentile for age and gender standards were considered as “underweight”, those between the 15th percentile to less than <85^th^ percentile as “normal weight”, between the 85^th^ percentile to less than <95^th^ percentile as “overweight”, and equal to or greater than ≥95 was considered as “obese” (21). To determine these cut off points, we used the data on anthropometric parameters of all participants of TLGS, aged10-16 years. 

Lipid measurements including Total Cholesterol (TC), Triglycerides (TG) and High Density. Lipoprotein Cholesterol (HDL-C) were determined by commercial assay kits (Pars Azmoon Inc., Tehran, Iran), TC and TG were assayed using enzymatic colorimetric tests with cholesterol esterase and cholesterol oxidase, and glycerol phosphate oxidase, respectively. HDL-C was measured after precipitation of the apolipoprotein B containing lipoproteins with phosphotungistic acid. All samples were analyzed when internal quality control met the acceptable criteria. In all the biochemical analyses, the intra- and inter-assay coefficients of variation (CV) were less than 2.5% and 3.2%, respectively. Study participants were categorized according to their serum LDL-C values into three groups: 1) <110 mg/dl; 2) between 110-129 mg/dl and 3) >129 mg/dl. Based on their serum cholesterol study participants were placed in three groups: 1) <170 mg/dl, 2) between 170-199 mg/dl and 3) >199 mg/dl (20, 22, 23).

**Statistical analysis**

Continuous variables, checked for normality, using the one-sample Kolmogorov-Smirnoff test, are expressed as mean±SD deviation. Logarithmic transformation was applied to not normally distributed variables. Categorical variables are expressed as percentages. 

The association between age at menarche with anthropometric parameters and lipid profiles with normal distribution was assessed using the Pearson correlation coefficient. Distributions of age at menarche between BMI, LDL-C and cholesterol groups were compared, using analysis of variance. Data analysis was performed using the SPSS 15.0 PC package (SPSS Inc., Chicago, IL). P≤0.05 were considered as significant.

## Results

Of 727 girls, aged 10-16 years, 370 met our inclusion criteria. The mean±SD for age of menarche, weight, height, waist circumference and body mass index were 12.6±1.1 years, 53.7±10.5 kg, 157.3±5.5 cm, 72.5±9.8 cm and 21.7±3.9 kg/m^2^, respectively. 

**Figure 1:**
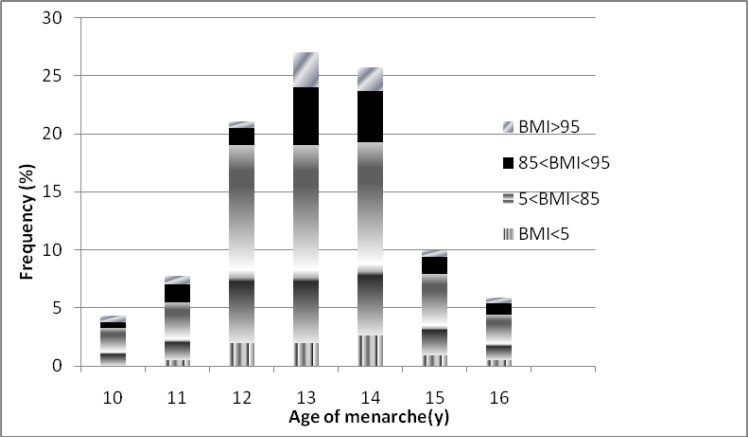
Distribution of age at menarche according to BMI groups

The menarche age range of 11-13 years constitutes 80.5% of distribution. General characteristics, anthropometrics and lipids parameters of study participants are demonstrated in table I. Prevalences of under, normal and overweight girls were 4.3%, 72.4% and 11.9%, respectively. Of our study participants, 11.4% were obese. Menarcheal age distribution of the study subjects, according to the BMI categories is shown in figure 1. There was a negative correlation between BMI and age at menarche using regression linear analysis (p=0.004, R-square=0.02). 

Although, age at menarche had a statistically significant positive correlation with height (R-square=0.04, p<0.005), it had a statistically significant negative correlation with waist circumference (R-square=0.01, p=0.04). No statistically significant relationship was found between menarcheal age and weight, waist hip ratio (WHR) and lipids parameters (R-square=0.00, p=0.9) (Table II).

No significant correlation was seen between LDL groups and menarcheal age after adjustment for BMI, height, weight, waist circumference, HDL cholesterol and total cholesterol. The influences of various independent variables, including mother’s educational status, anthropometric parameters and lipids profiles on age at menarche were explored using the multiple regression analysis (stepwise method). 

There was a statistically significant parameters correlation between age at menarche and the mother’s educational status, with increasing mother’s education, age of menarche decreased (β=-0.112, CI=-0.22, -0.003, p=0.04), age of menarche increased as BMI decreased (β=-0.110, CI=-0.181, -0.04, p=0.005). 

**Table I:** The basic and biochemical characteristics of study subjects

**Variables**	**Value**
Age of menarche (years)	12.6±1.1
Weight (Kg)	53.7±10.5
Height (cm)	157.3±5.5
BMI (Kg/m^2^)	21.7±3.9
Waist circumference (cm)	72.5±9.8
Waist hip ratio	0.2±0.01
Total cholesterol (mg/dl)	151±24.9
Triglycerides* (mg/dl)	4.5±0.4
High-density lipoprotein (mg/dl)	39±9.1
Low-density lipoprotein (mg/dl)	92±22.7

Value (means±SD).

* SD of log-transformed.

**Table II:** The relationship between age of menarche and anthropometric indexes and serum lipoproteins

**Variables**	**β**	**p-value**
Weight	-0.07	0.18
Height	0.21	0.00*
BMI	-0.16	0.004*
WC	-0.1	0.04*
WHR	-0.001	0.9
Total cholesterol	0.07	0.2
Triglycerides **	0.008	0.8
High-density lipoprotein	0.00	0.9
Low-density lipoprotein	0.07	0.22

β=Regression coefficient.

* p<0.05.

## Discussion

Our study demonstrated no significant relationship between age of menarche and lipid parameters after adjustment for other anthropometrics and socio-economic variables. We did however find a negative association between age at menarche and body mass index. The mean menarcheal age of our study cohort was 12.6±1.1 years and the onset of menarche in majority of our subjects (80.5%) was between 11-13 years of age, similar to that reported by Khabazan *et al* in Tehran (24). The mean ages of menarche among Iranian girls reported in two other studies (1990, 1999) were 13.86 and 13.65 years respectively (25). 

The mean±SD height of girls in this study was 157.3±5.5 cm. There was a significant positive correlation between the menarcheal age of our study subjects and their height, results similar to those demonstrated by Elizando (26) and Kabir *et al* (27). Various studies have documented contradictory results regarding the effect of weight on menarcheal age. In our study, there was no significant statistical relationship between weight and age of menarche, results similar to those reported in another study (24). Our results were in contrast with those of Meyer *et al* (28) and the Kim *et al* study (29) that found a positive relationship between weight and menarcheal age. In a study from Peru, an inverse relationship was observed between weight and body mass index and menarche age (30). 

The impact of BMI on age at menarche is not clear yet. Merzenich *et al* reported that reaching a critical weight of 47.8 kg is a requisite for occurrence of menarche in girls (31); however studies have reported various associations between ages at menarche with BMI. In the present study, similar to other studies (32, 33), menarcheal age reduced with increasing body mass index. Other studies however found no significant association between BMI and average age of menarche (10). Kapiro *et al* found a linear association between BMI and menarche age in twin girls (9), whereas Demerath *et al* reported that onset of menarche and changes in body mass index were independent (34). There are studies that report a relationship between menarche and adiposity; the age at menarche of those girls with higher body mass index being less than others (18, 35-37).

There is limited data or associations between lipid profiles and menarcheal age (38-40). According to a study conducted in Spain on 272 girls aged 9-15 years, no significant association was found between the menarche age and blood lipids or waist circumference (39), very similar to results reported by Tell *et al* (40). Also, Kim *et al* study showed that there is a positive association between age at menarche and waist to hip ratio and waist circumference (29). Our study demonstrated no significant relationship between age of menarche and lipid parameters after adjustment for other anthropometric and socio-economic variables. Maternal educational level had a significant effect on menarcheal age in our study, which is in agreement with those reported by Kurdzielewicz in a study of 111 girls in Netherland (19).

There are several explanations for the various factors that affect age at menarche; these include the socio-economic and nutritional status and environmental conditions as well as genetic determinants. (2-10, 37, 41-43). Furthermore the recruitment of study subjects and the type of study have a great impact of the age at menarche and its other influencing factors (1, 17, 44-46). Our study has the advantage of examining this issue in a population-based cohort of Iranian girls with a specific age range. Furthermore, the amount of intra-assay variability in our data is likely to be minimal, because all the laboratory measurements were evaluated in the same laboratory by the same person. 

Of our few limitations, the most significant was that we did not use a standard method for identification of the fat mass. Also we did not evaluate the effect of other important variables such as physical activity or nutritional status on menarcheal age. It needs to be mentioned that the current analyses were based on data from a single community in Tehran as a result it may not be possible to generalize these results to other communities in the Islamic Republic of Iran. In conclusion it seems that age at menarche is not influenced by lipid profiles.
